# Critical upward shift of intracranial pressure levels in extremely obese patients; normalization due to bariatric surgery

**DOI:** 10.1186/s13089-025-00439-z

**Published:** 2025-07-28

**Authors:** Nabil Al Shammas, Robert Luck, Sophie Schumann, Dragana Köhler, Lutz Mirow, Bernhard Rosengarten

**Affiliations:** 1 Department of Neurology, Chemnitz Medical Center, Flemmingstraße 2, 09116 Chemnitz, Germany; 2 Department of General and Visceral Surgery, Chemnitz Medical Center, Campus of the Medical Faculty of the Technical University Dresden, Flemmingstraße 2, 09116 Chemnitz, Germany

**Keywords:** Obesity, Transcranial Doppler, Intracranial hypertension, Non-invasive intracranial pressure, Bariatric surgery

## Abstract

**Background:**

Increase in body mass index (BMI) is a risk factor for idiopathic intracranial hypertension (IIH). The matter of body weight and intracranial pressure (ICP) in clinically asymptomatic obese patients is unknown. We aimed at studying the relationship of ICP and BMI pre- and post-surgery in obese patients undergoing bariatric surgery.

**Methods:**

Patients with a BMI > 35 kg/m^2^, qualified for bariatric surgery and without clinical signs of IIH were prospectively and consecutively included. The optic nerve sheath diameter (ONSD) and a combined transcranial Doppler-arterial blood pressure (TCD&ABP-ICP) method were used to non-invasively determine the ICP (nICP) pre- and post-surgery (six months after surgery when weight loss had stabilized). ONSD > 5.8 mm and nICP > 25cmH_2_O were assumed as pathologically increased. A nICP between > 20 and ≤ 25 cmH2O was assumed as being in the borderline.

**Results:**

54 patients (43 female; 44 ± 11 years old) were included. Pre-surgery BMI (46 ± 6 kg/m^2^) significantly declined after surgery (post-surgery BMI: 32 ± 6 kg/m2; paired t-test: *p* < 0.0001). Initial ONSD was 5.8 ± 0.6 mm (6 pathological values) which declined to 5.4 ± 0.6 mm (5 pathological values) (paired t-test: *p* < 0.025). TCD&ABP assessed nICP was 19 ± 4.5 cmH_2_O (5 with pathological, 16 with borderline values) pre-surgically and declined to 14 ± 4 cmH_2_O (no pathological, 1 high-normal value) after surgery (*p* < 0.0001).

**Conclusion:**

Assuming the low incidence of IIH, the frequency of pathologic and borderline ICP values in obese patients was unexpectedly high. Reduction of ICP with weight loss followed a simple regression line pointing to a mechanistic effect of increased body weight on ICP. The constancy of pathologic ONSD values might be due to a fixed dilatation of the optic nerve sheath due to the duration of obesity.

## Introduction

Idiopathic intracranial hypertension (IIH) is considered as a rare disease which usually affects young obese women in child bearing age [1]. However, due to increasing obesity rates in population the incidence of IIH is rising with an incidence of about 10/100,000 per year [2, 3]. Above a body mass index (BMI) of 30 kg/m^2^ the incidence of IIH increases in women and men [3–5], although even a modest weight gain increases risk of developing IIH [4, 6].

IIH is characterized by raised intracranial pressure (ICP), resulting in headaches, dizziness, visual disturbances and even blindness due to papilledema [7]. Psychiatric disorders are sevenfold more common in IIH patients compared with the general population [6, 8]. Obese patients with IIH also suffer from cognitive deficits [9–11], from which Grech et al. found that the executive cognitive function is typically affected [11]. Interestingly, the authors did not only find cognitive deficits in the patient’s group but also in presumably “healthy” participants of the body weight matched control group. A lumbar puncture with liquor drainage led to a rapid normalization of cognitive deficits in patients as well as affected control group participants. Weight loss due to bariatric surgery was also effective but it took several weeks until ICP and therefore cognitive dysfunction normalized [11].

Although the lumbar puncture still is the gold standard for determining increased ICP in this group of patients [7], its invasiveness and sometimes painful execution limits its use in asymptomatic patients. Non-invasive techniques can overcome the disadvantages of lumbar puncture. A duplex-sonography based measuring of the optic nerve sheath diameter (ONSD) as well as a combined transcranial Doppler- arterial blood pressure technique (TCD&ABP) based non-invasive intracranial pressure (nICP) assessment were repeatedly used in several IIH related studies to assess the ICP non-invasively [12, 13].

We aimed to study ICP levels non-invasively in extremely obese patients (BMI ≥ 35 kg/m^2^) without clinical signs of an IIH. In addition, the effect of bariatric surgery on BMI and ICP values was aim of our study. We hypothesize that obese patients have higher ICP levels, which decline with decreasing BMI values after bariatric surgery.

## Materials and methods

From 2023 to 2024 we prospectively and consecutively included obese patients for bariatric surgery in our study. Patients aged between 18 and 55 years and with a BMI ≥ 35 kg/m^2^ were included, who had failed to lose weight or maintain weight loss. We obtained ONSD and TCD&ABP assessed nICP before surgery (pre-surgery) and after more than 6 months when weight loss has stabilized (post-surgery). No patient had a diagnosis of IIH or had clinical signs of IIH according to the recommendation of Mullan et al. [14]. All patients underwent a Roux-en-Y gastric bypass surgery, which was the most successful weight loss method [15]. Body heights and body weight of patients was recorded to calculate the BMI.

ONSD was assessed with B-mode using a Philips iU22 ultrasound system and a 9 − 3 MHz linear array transducer (Philips Medical Systems; Bothell, WA, USA). Examinations were done in a supine position with the upper part of the body and the head elevated to 20–30°. The mechanical index (MI) was reduced to 0.2, the thermal index to 0.0. The ultrasound probe was placed on the closed upper eyelid using ultrasound gel. The anterior part of the optic nerve was searched in a transversal plane showing the papilla and the optic nerve in its longitudinal course. ONSD was assessed 3 mm behind the papilla, as described previously [12]. ONSD was obtained once as maximal diameter of the outer limits of the optical nerve sheaths and was obtained for the right and left side and the mean value was used for further evaluation. Due to reference values of our neurophysiologic laboratory, values above 5.8 mm were assumed as pathologic [16].

TCD&ABP related nICP was assessed in a supine position on a comfortable diagnostic chair. The cerebral blood velocity was assessed by transcranial Doppler (TCD) using a 2-MHz pulsed Doppler monitoring probe (Delica EMF-9 d pro, Shenzen Delica Medical Equipment Co., China). Blood velocity was obtained from both middle cerebral arteries (MCAv) in a depth of about 55–65 mm. TCD probes were secured in place by using a headset provided by the device manufacturer. The arterial blood pressure (ABP) was continuously and non-invasively measured with a photoplethysmographic cuff method (Finapres NOVA, Finapres Medical Systems BV, Enschede, The Netherlands), placed around a finger. The calibration sensor for the ABP was placed at MCA level. MCAv and ABP data were streamed to a windows laptop where the ICM + software (ICM+, Cambridge Enterprise, University of Cambridge, UK) could collect and integrate the data at 1 kHz [17]. A nICP software plugin within ICM + was used to calculate nICP, as previously reported [13, 18]. In short: the intracranial compartment is considered a black-box system, with ICP being a system response to the incoming signal ABP. This mathematical model provides a method to describe the transmission characteristics, with input and output signals. The intracranial compartment is modelized by a so-called impulse response function which connects the assumed input signal, ABP, with the output signal, ICP. Then, two linear models are established to depict the relationship between ABP and ICP (ABP→ICP model) and the relationship between ABP and MCAv with the application of certain TCD characteristics such as peak systolic, enddiastolic flow velocity and steepness of flow velocity increase and decrease, see for more detail [10]. The TCD characteristics may be derived from ABP and MCAv signals and, therefore, can be assessed noninvasively from the patient. The essential part of our nICP procedure is a description of the relationship between the TCD characteristics and the ABP → ICP model. A signal database including invasively assessed ICP of reference patients was used for this purpose. Therefore, the ABP → ICP model can be calculated from TCD characteristics, and its output data provides a continuous nICP waveform. From a 10-minute recording the data was obtained during a stable phase of parameters [13]. nICP values were averaged over a beat-to-beat calculation of 20 heart beats and averaged from both sides.

Whereas nICP levels above 25 cmH2O were assumed as pathological, levels between 20 and 25 cmH2O were assumed as being in the borderline range. Values below 20cmH2O were assumed as normal values.

### Statistics

For evaluations we used statistical software (StatView, Version 5.0.1., SAS Institute, North Caroline, USA). Data were given as mean ± standard deviation. Normal distribution was tested by Shapiro-Wilk test. Pre-surgery vs. post-surgery data were evaluated by a paired t-test in the case of normal distribution, otherwise the Mann Whitney U test was used. Test results with a probability *p* < 0.05 were considered significant.

Pre-surgery and post-surgery ONSD and nICP data were plotted separately (y-axis) against the BMI values (x-axis) and a linear regression analysis with the least-square method was conducted according to Berdahl et al. [19].

## Results

**Fig. 1:**
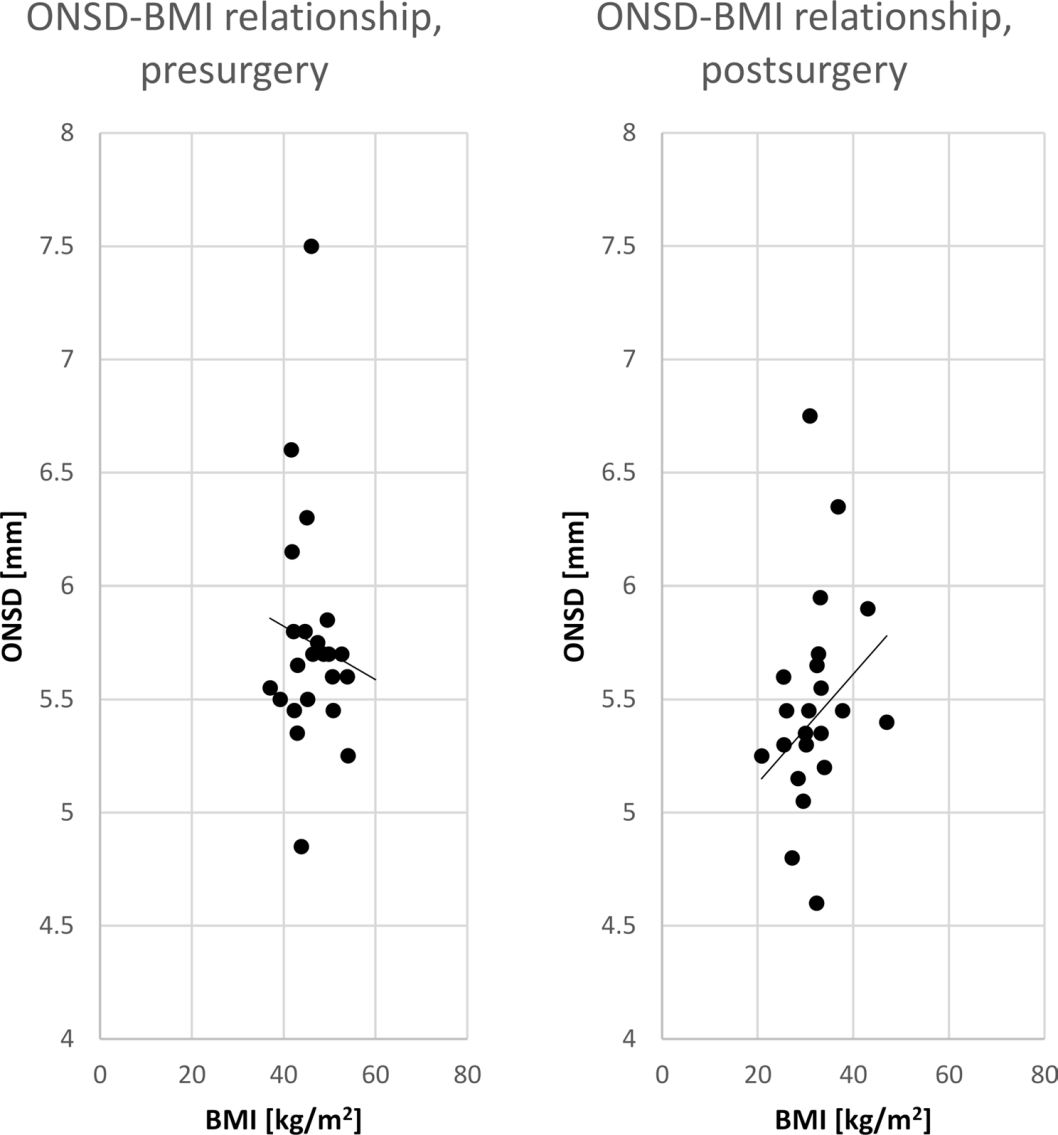
ONSD vs. BMI. Data are given for pre- (left side) and post-surgery (right side) conditions. With reduction of the BMI the mean ONSD fell from 5.8 ± 0.6 to 5.4 ± 0.6 with a significance level of *p* < 0.025. In addition, the regression line is given for both conditions. ONSD: optic nerve sheath diameter; BMI: body mass index

**Fig. 2:**
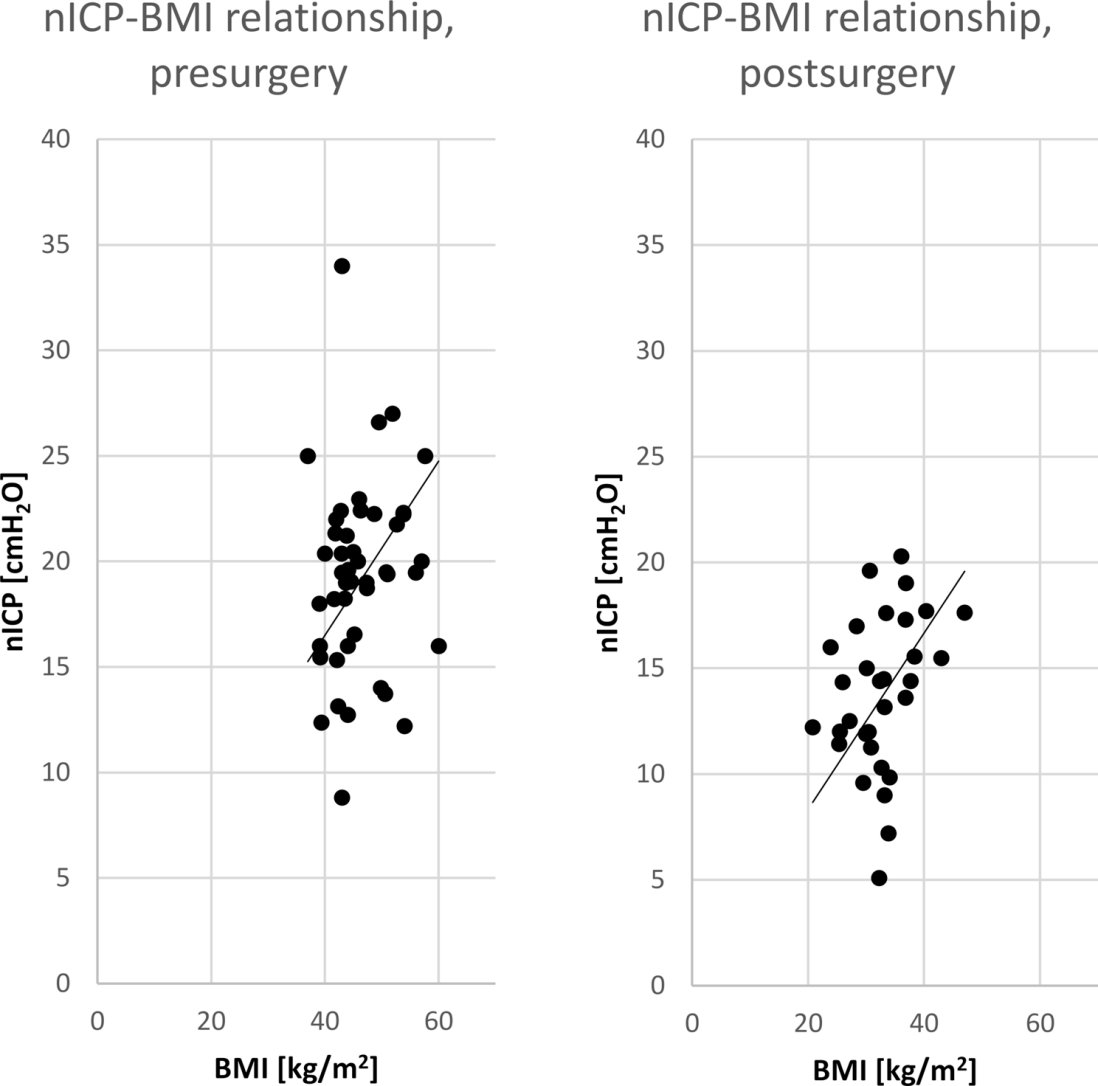
nICP vs. BMI. Data are given for pre- (left side) and post-surgery (right side) conditions. With reduction of the BMI the mean nICP decreased from 19 ± 4.5 to 14 ± 4 with a significance level of *p* < 0.0001. In addition, the regression line is given for both conditions. nICP: non-invasive intracranial pressure; BMI: body mass in

**Fig. 3:**
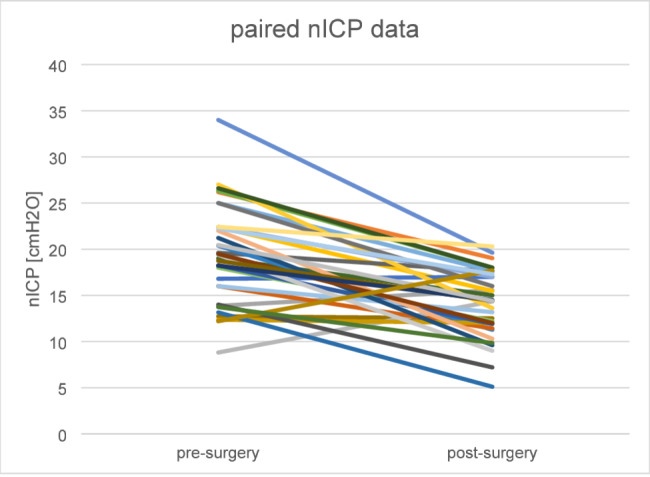
Paired nICP data. Pre- and postinterventional data pairs for each individual. With reduction of BMI the increased nICP levels decreases

We included 54 patients (43 female) with a mean age of 44 ± 11 years. Clinically, no patient had signs of IIH. BMI, ONSD and nICP results pre- and post-surgery are shown together with statistical test results in Table 1. The individual pre- and post-surgery data for the relation of BMI and ONSD are given in Fig. 1, whereas Fig. 2 shows the data for nICP. The BMI-ONSD regression coefficient R^2^ was 0.08 pre-surgery and 0.06 post-surgery. The BMI-nICP regressions coefficients were 0.99 and 0.94, respectively. The paired changes in nICP values pre- and post-surgery are given in Fig. 3.

The mean values of ONSD and nICP were formally in the normal range. However, 6 patients (4 female; 6.7 ± 0.6 mm) in the pre-surgery condition had an ONSD ≥ 5.8 mm, from which one normalized post-surgery (5.7 mm). 5 patients (4female; 28 ± 3 cmH_2_O) in the pre-surgery condition had a pathologically increased nICP ≥ 25 cmH_2_O, whereas no patient had an increased nICP ≥ 25 cmH_2_O post-surgery. Pre-surgery 16 patients were in the borderline range (nICP ≥ 20cmH_2_O and < 25cmH_2_O), whereas 1 patient had an nICP = 20,3 cmH_2_O post-surgery. Under pre-surgery conditions 4 patients had both an increased ONSD as well as nICP. After surgery, only one patient with borderline nICP matched with an increased ONSD.

**Table 1:** Primary test results; BMI, nICP, ONSD changes at pre- and post-surgery conditions

	Pre-surgery	Post-surgery	Paired t-test
BMI [kg/m^2^]	46 ± 6	32 ± 6	*p* < 0.0001
nICP [cm H_2_O]	19 ± 4,5	14 ± 4	*p* < 0.0001
ONSD [mm]	5.8 ± 0.6	5.4 ± 0.6	*p* < 0.025

BMI: body mass index; nICP: non-invasive intracranial pressure; ONSD: optic nerve sheath diameter

## Discussion

In the present study we showed that the mean ONSD and nICP values significantly decline with weight reduction of obese patients. The nICP values show more clearly the normalization, possibly because they express pressure values more accurately than the ONSD data. Also, the regression analysis with combined data from pre-surgery and post-surgery conditions showed a higher coefficient of determination between BMI and nICP values as compared to the coefficient between BMI and ONSD data. The regression line of the association between BMI and nICP values matches excellently with a previous study from Berdahl et al. who correlated BMI data with lumbar puncture pressure results from more than 4000 patients [19]. These data support our assumption of a general linear relationship between BMI and ICP and are in line with mechanistic concepts of a relation between BMI and ICP: an increase in truncal body mass results in an increased intrathoracic and therefore increased central venous pressure, which in consequence increases intracranial pressure [15]. Similarly, ICP increases when the end-expiratory pressure in artificially ventilated patients is increased [20], or abdominal insufflation for laparoscopy takes place [21]. Zunino et al. found a nearly linear correlation between changes in intraabdominal pressure and the ICP, which was supported by data from animal experiments [21, 22]. Negative abdominal pressure reduces ICP followed by a rapid relieve of symptoms in IIH patients [23]. Nevertheless, other mechanisms related to obesity such as an altered hormone level, increased leptin levels or obstructive sleep apnea might also affect liquor production and absorption [24].

Approximately 10% of patients had a pathologically increased ONSD or nICP, and nearly 30% of patients had a borderline increased nICP. These numbers are of concern, as they are much higher than the expected incidence rates of IIH in the general population. We therefore support reports that extreme obesity significantly increase the risk for developing IIH. Normalization of ICP after weight reduction points to a reversible syndrome and therefore the question arises, if extremely obese patients should be screened routinely with non-invasive methods for existence of an increased ICP. The present patients with increased nICP values were formally asymptomatic regarding clinical signs of IIH. Further studies have to be undertaken in which patients with increased nICP values are controlled by lumbar puncture and in which more sophisticated neuropsychological tests such as used by Grech et al. are performed to address for side effects of increases in ICP [11]. Also, ICP lowering agents such as acetazolamide, furosemide or topiramate should be studied if they can relieve increased ICP levels in obese patients. Furthermore, when increased ICP values persist after drainage or under medication patients should be recommended for earlier bariatric surgery instead of dietary programs.

A limitation of this study in “healthy” obese patients was, that we could not use the invasive lumbar puncture method due to ethical reasons. Lumbar puncture is still assumed to be the gold standard to assess an increased ICP in IIH [14]. Another issue which should be addressed in further investigations is the lacking correlation between BMI and ONSD values in the pre-surgery situation. The lacking correlation in the higher BMI range might point to a so called “ceiling effect” in which the diameter of the ONSD reaches a maximum despite further increasing levels of BMI. Under reduced BMI values after surgery and weight loss the relation between ONSD and BMI seemed to re-establish but on a low correlation niveau. Although the mean ONSD values decrease from pre-surgery to post-surgery conditions, a clear individual association cannot be seen in our data. It might be speculated if the dilated ONSD might be at least in part irreversible. In patients with acute occurrence of IIH we found a reversible ONSD dilation with normalization of increased ICP levels [12]. A further limitation might be the classic concept of absolute pressure levels to decide between normal and pathologically increased ICP values. Alternatively, a transitive concept between normal and increased ICP levels would have been more appropriate in the present investigation. A transitive concept might help to explain why apparently clinically healthy patients have increased ICP levels or present with subtle cognitive deficits as shown by the study of Grech et al. [11].

## Conclusion

Increased body mass results in an upward shift of nICP levels with approximately 30% in the borderline and 10% in the pathological range. Bariatric surgery with a Roux-Y bypass does not only effectively reduces BMI but also relieves from pathologically increased nICP levels.

## Data Availability

Owing to local privacy policy conditions data are not publicly available. In case of interest a request should be sent to the corresponding author.

## Declarations

### Patient and public involvement

This study did not involve patients or public issues.
